# Characterization of Histone Modifications Associated with Inactive X-Chromosome in Trophoblast Stem Cells, eXtra-Embryonic Endoderm Cells and in *In Vitro* Derived Undifferentiated and Differentiated Epiblast Like Stem Cells

**DOI:** 10.1371/journal.pone.0167154

**Published:** 2016-12-15

**Authors:** Cathérine Dupont, Cheryl Maduro, Hannah Den Braanker, Ruben Boers, Dorota Kurek, Joost Gribnau

**Affiliations:** Erasmus MC, Department of Developmental Biology, Rotterdam, The Netherlands; Michigan State University, UNITED STATES

## Abstract

In mouse, X-chromosome inactivation (XCI) can either be imprinted or random. Imprinted XCI (iXCI) is considered unstable and depending on continuous *Xist* expression, whereas random XCI (rXCI) is stably maintained even in the absence of *Xist*. Here we have systematically examined epigenetic modifications associated with the inactive X-chromosome (Xi) in Trophoblast Stem cells, eXtra-Embryonic Endoderm Cells, undifferentiated and differentiated Epiblast Like Stem Cells in order to understand intrinsic differences in epigenetic mechanisms involved in silencing of the inactive X-chromosome in lineages presenting iXCI and rXCI. Whereas euchromatic histone modifications are predominantly lost from the Xi territory in all cell types, the accumulation of heterochromatic modifications diverges in between the analysed cell lineages. Particularly, only the Xi of multipotent Trophoblast (iXCI) and Epiblast stem cells (rXCI) display a visible accumulation of Polycomb Repressive Complexes (PRCs), in contrast to the Xi in differentiated Epiblast Like Stem Cells and eXtra-embryonic Endoderm cells. Despite this, the histone modifications catalysed by PRCs, ubH2AK119 and H3K27me3, remain the best heterochromatic markers for the Xi in all assessed lineages. Heterochromatic chromatin modifications associated with the Xi are a reflection of the epigenetic landscape of the entire genome of the assessed cell regardless whether XCI is imprinted or random.

## Introduction

Sex determination in mammals is determined by the presence of the male sex determining gene SRY located on the Y-chromosome. Female mammals have two X-chromosomes, whereas males possess one X-chromosome and one Y-chromosome. This imbalance is compensated through inactivation of one of the two X-chromosomes in all female cells.

The actual silencing of the X chromosome in placental mammals is a highly dynamic and complex process. A crucial player in initiation of silencing of the X is the long non-coding RNA *Xist* [1,2]. *Xist* RNA recruits specific protein complexes, which trigger, a cascade of epigenetic events resulting in the inactivation of the *Xist-*expressing X-chromosome [3]. A widely used animal model to study X-inactivation (XCI) is the mouse. In the female mouse embryo, *Xist* starts to be expressed during early embryogenesis from the 2-cell stage onwards, leading to silencing in cis. This form of XCI is referred as imprinted XCI (iXCI), as it exclusively leads to XCI of the paternally derived X-chromosome. Whereas all developing extra-embryonic lineages maintain iXCI, lineages that will form the embryo proper characteristically erase iXCI and re-establish XCI in a random manner (rXCI) [4]. *In vitro* differentiation of embryonic stem (ES) cells derived from the inner cell mass (ICM) has provided quite detailed information on the sequence of epigenetic events assisting in the inactivation of one of the X-chromosomes in embryonic tissues [5,6,7,8,9,10,11]. In differentiating ES cells the first epigenetic event following the accumulation of *Xist* is the loss of euchromatic marks such as methylation of histone H3K4 and acetylation of H3K9. Subsequently, characteristic repressive histone modifications like methylation of H3K27, H3K9 and H4K20 and ubiquitination of H2A can be detected on the Xi. XCI in extra-embryonic tissues is, in contrast to fully differentiated embryonic tissues, considered unstable [12,13,14,15,16]. In order to understand how and why XCI is stable or unstable and if epigenetic events differ between rXCI and iXCI, a full characterization of chromatin modifications in lineages of differing origin is necessary.

In this study, we have systematically characterized histone modifications associated with the inactivated X-chromosome (Xi) in trophoblast stem (TS) cells, eXtra-embryonic Endoderm (XEN) cells, *in vitro* derived Epiblast Like Stem Cells (EpiLCs) and to mesoderm differentiated EpiLCs. The obtained data were completed with reported data of chromatin modifications on the Xi in pre-implantation embryos (Table 1) and cell lineages directly derived from the pre- and early post-implantation embryo (Table 2). This study has generated a comprehensive overview of the epigenetic landscape of the Xi in different cell lineages presenting either iXCI or rXCI.

**Table 1:** Chromatin Marks associated with the Xi in pre-implantation embryos.

Chromatin Marks Xi	4-cell	8-cell	Morula	Blastocyst
H3K27me3		Absent [17,18]	Present [17,18] Absent [6]	Present [17,18] Present [6]
H4K20me1				
H3K9me2		Absent [17,18]	Absent [17,18]	Present [17,18]
ubH2A				
Polycomb Repressive Complex 1				
Polycomb Repressive Complex 2		Absent [17,18]	Present [17,18] Absent [6]	Present [17,18]Present [6]
RNA Polymerase II exclusion	Present [17,18]	Present [17,18]	Present [17,18]	Present [17,18]
H3K4me2 exclusion	Absent [17,18]	Present [18];	Present [17,18]	Present [17,18]
H3K9ac exclusion	Absent [17,18]	Present [18]	Present [17,18]	Present [17,18]
H4ac exclusion			Present [17,18]	Present [17,18]
H4K16Ac exclusion				

**Table 2:** Chromatin Marks associated with the Xi cell lineages.

Chromatin Marks Xi	ES Differentiation	MEF	TS Cell	Trophoblast and differentiated TSC	XEN
H3K27me3	Transient [5,6,7]	Absent [6]	Present [5,6,19,20]	Transient [5,6] Stable [14]	Present [21] Absent [19,20]
H4K20me1	Transient [7]		Present [19]	Transient [14]	
H3K9me2	Stable [7,8,9,10] Absent[7]	Stable [8] Absent [7]	Minorly present [20]		Minorly present [20]
ubH2A	Transient [5]		Present [5,19]	Transient [5]	
Polycomb Repressive Complex 1	Transient [5]		Present [5,19]	Transient [5]	
Polycomb Repressive Complex 2	Transient [5,6,7]	Absent [6]	Present [5,6,19,20,22]	Transient (Ezh2 and Eed1) [5,6] Stable (Eed1) [14]	Absent [19,20]
RNA Polymerase II exclusion	Stable [11]			Absent to partially present [14]	
H3K4me2 exclusion	Stable [7,8,9]	Present [8]	Present [19,20]	Minorly present [14]	Present [20]
H3K9ac exclusion	Stable [8,9]	Present [8]	Present [20]		Present [20]
H4ac exclusion	Stable [8,9,23]	Present [8]	Present [19]	Absent to partially present [14]	
H4K16Ac exclusion	Stable [9]			Absent [14]	
H3Ac exclusion			Present [19]		

## Results

Despite the wealth of experiments, a complete and comprehensive overview of all histone modifications associated with the Xi in cell types of different embryonic lineages is lacking. We therefore generated TS, XEN, and ES cells from pre-implantation embryos with the same genomic back ground, and differentiated the ES cell lines into EpiLCs, that were further differentiated towards the mesodermal lineage using WNT3 and BMP4 ligands. For our studies we examined Xi and *Xist* associated histone modifications in extra-embryonic TS and XEN cell lines, and in undifferentiated and differentiated EpiLCs with an embryonic origin. The obtained results were compared to available data in the literature (reviewed in Tables 1 and 2).

### Loss of euchromatic marks on the Xi

**Fig 1:**
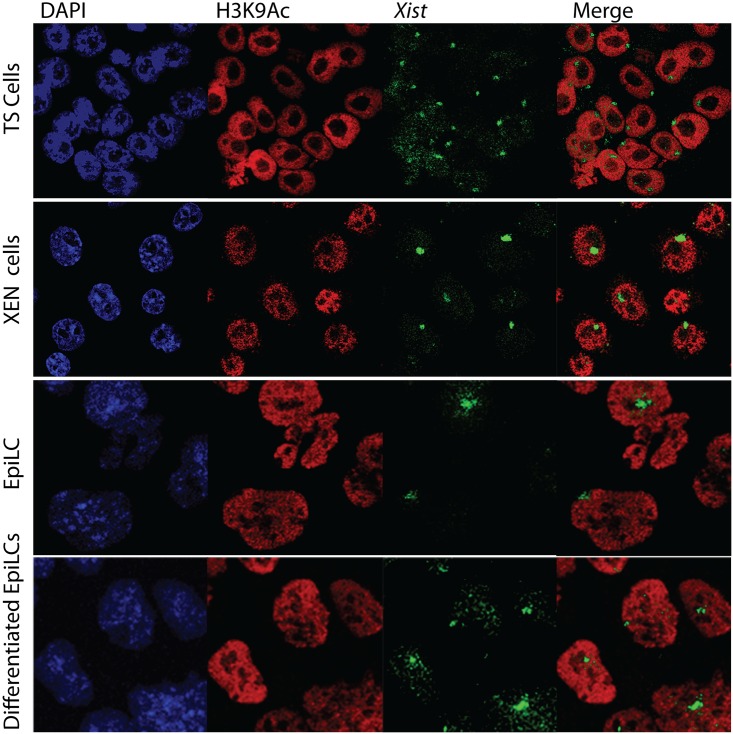
Loss of euchromatic mark H3K9Ac on Xi in TS cells, XEN cells, EpiLCs and differentiated EpiLCs. Immuno-RNA FISH detecting H3K9Ac (Rhodamine red) and *Xist* (FITC) on TS cells, XEN cells, EpiLCs and differentiated EpiLCs.

**Fig 2:**
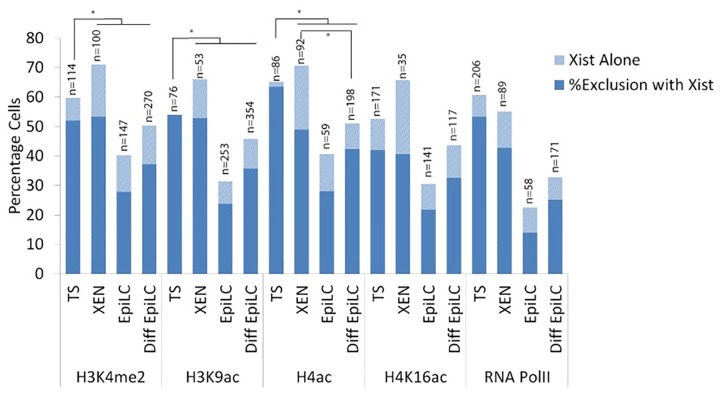
Percentage cells accumulating either *Xist* alone or showing *Xist* together with exclusion of euchromatic marks in TS cells, XEN cells, EpiLCs and differentiated EpiLCs. Percentages displayed in bar chart with sample size (n) above. Statistical significance (p<0,05) tested via z-test for proportion independent groups and shown in Figure.

**Fig 3:**
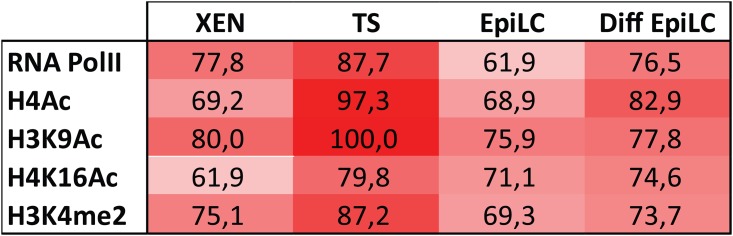
Percentage cells presenting exclusion of euchromatic modifications along *Xist* in XEN cells, TS cells, EpiLCs and differentiating EpiLCs. Percentage illustrated by a colour scale from no (blue) to complete (red) exclusion of euchromatic modifications from the Xi territory.

Previous studies indicate that the first epigenetic changes observed on the *Xist* coated X are related to loss of histone modifications, H3K4me2, H3K9ac, H4ac, H4K16ac and RNA polymerase II, all associated with active chromatin. To test whether these markers were depleted throughout our panel of cell lines we performed RNA FISH for *Xist* RNA in combination with immunohistochemistry for these histone modifications on TS (S1 Fig), XEN cells (S2 Fig), EpiLCs (S3 Fig) and differentiated EpiLCs (S4 Fig). To quantify the results, 53 to 354 cells were counted and the percentage of cells displaying *Xist* clouds with and without co-localization of lost euchromatic marks was determined (Figs 1 and 2). Although the detection varied per cell type, loss of euchromatic marks is a feature that is present in a high percentage of cells in all lineages, indicating that the loss of euchromatic marks is detected in lineages that are both independent (differentiated EpiLCs) and fully dependent on *Xist* expression (TS and XEN) for maintenance of XCI (Fig 3).

### Polycomb repressive complexes

**Fig 4:**
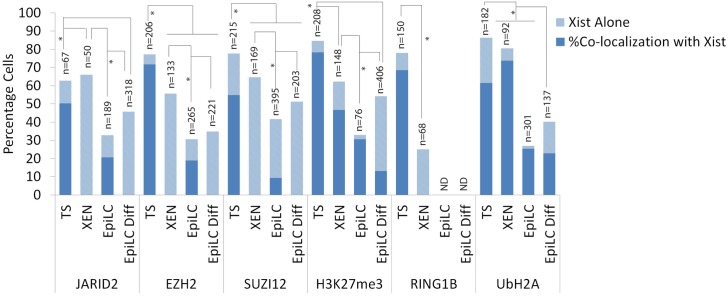
Percentage cells accumulating either *Xist* alone or showing *Xist* together with members of the PRC1 and 2 complexes and their catalysed modifications ubH2AK119 and H3K27me3 in TS cells, XEN cells, EpiLCs and differentiated EpiLCs. Percentages displayed in bar chart with sample size (n). Statistical significance (p<0,05) tested via z-test for proportion independent groups and shown in Figure.

**Fig 5:**
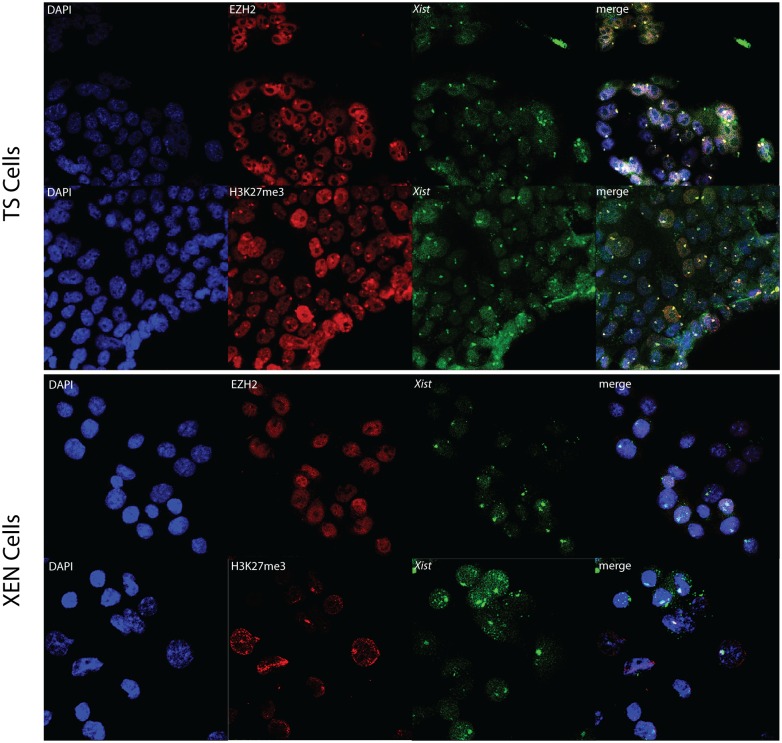
Immuno-RNA FISH for EZH2 or H3K27me3 and *Xist* in TS cells and XEN cells. Immuno-RNA FISH detecting EZH2 or H3K27me3 (Rhodamine red) together with *Xist* (FITC) on TS cells and XEN cells.

**Fig 6:**
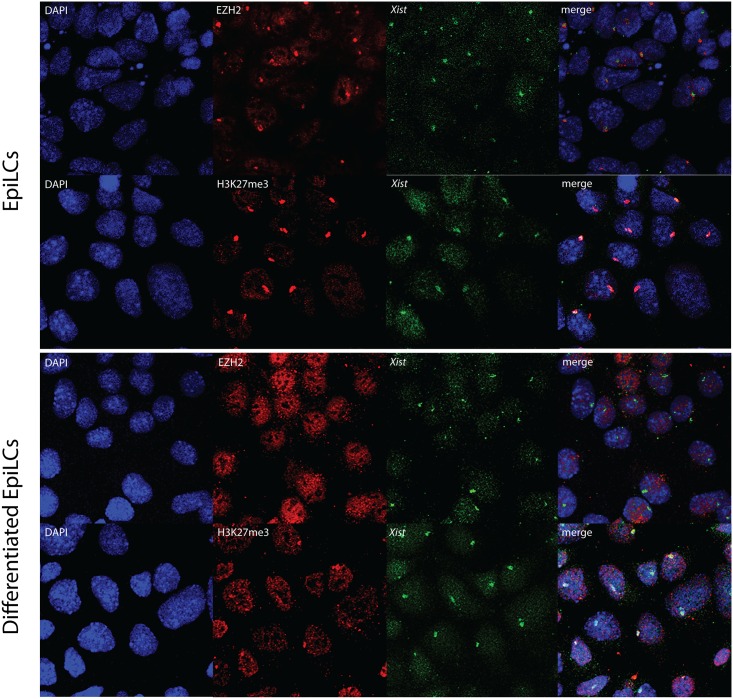
Immuno-RNA FISH for EZH2 or H3K27me3 and *Xist* in EpiLCs and differentiated EpiLCs. Immuno-RNA FISH detecting EZH2 or H3K27me3 (Rhodamine red) together with *Xist* (FITC) on EpiLCs and differentiated EpiLCs.

Silencing of the X chromosome is thought to proceed via the recruitment of polycomb repressive complexes (PRC) 1 and 2. While each complex consists of several proteins, for our studies only RNF2/RING1B has been assessed from the PRC1 complex, and JARID2, EZH2 and EED were evaluated as representative of the PRC2 complex, using the same panel of TS, XEN, EpiLC and differentiated EpiLC lines. RNA-immuno FISH was performed detecting *Xist* in combination with one of the PRC complex members. The number of cells counted per staining is displayed in Fig 4. As previously reported [5,6,19,20], TS cell lines distinctly displayed accumulation of PRC2 on the Xi (Figs 4 and 5, S5 Fig). Whereas PRC2 associated proteins to some extent also clearly accumulated on the Xi in undifferentiated EpiLCs, Xi associated accumulation of JARID2, EZH2 and SUZI12 was not detected in XEN cells or in differentiated EpiLCs (Figs 4, 5 and 6, S6, S7 and S8 Figs). JARID2 did show accumulation in XEN cells, but the accumulation was not associated with *Xist*. PRC2 catalyses trimethylation of lysine 27 on histone H3 (H3K27me3). Immuno-RNA-FISH analysis detecting H3K27me3 and *Xist* confirms that accumulation of this modification is present at the Xi in all cell types and indicates that even when the catalysing complex is below the detection limit, as found in XEN and differentiated EpiLC cells, its downstream modification is maintained (Figs 5 and 6). Nevertheless, the percentage of XEN cells and differentiated EpiLC cells with *Xist* clouds together with H3K27me3 accumulation is reduced compared to the other cell lines (Fig 4). The percentage of cells displaying an accumulation of H3K27me3 in differentiated EpiLCs was much higher when only immunostaining was performed. This was observed in all cell types studied and must be related to the more stringent conditions used for *Xist* RNA-FISH.

**Fig 7:**
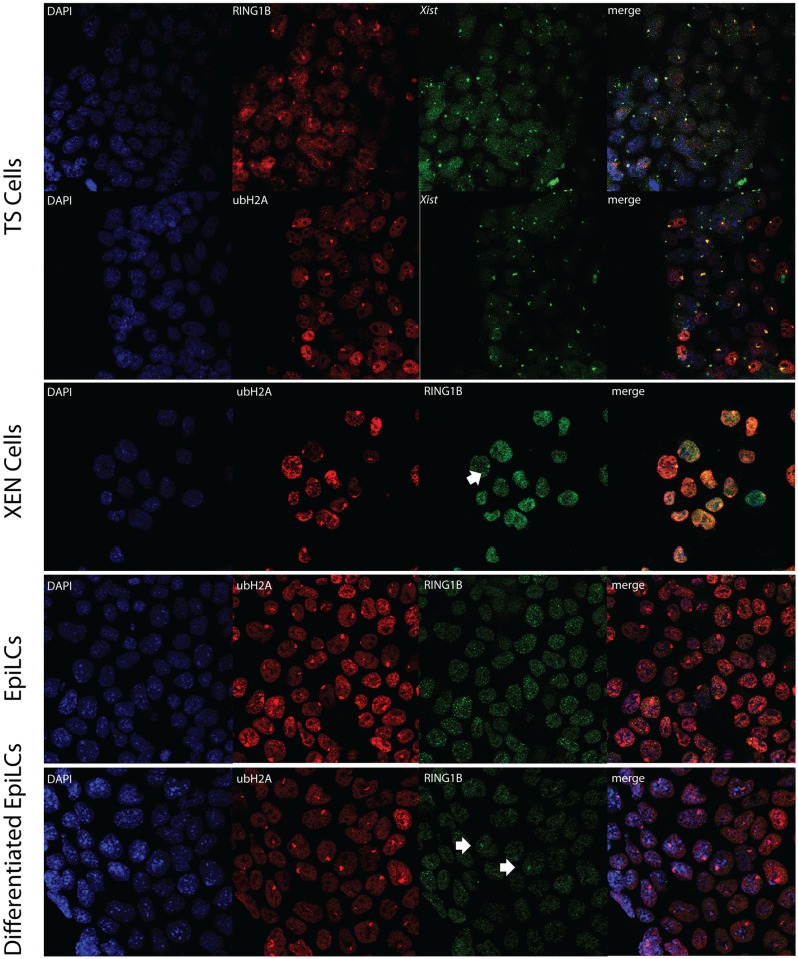
Immuno-RNA FISH for ubH2AK119 and *Xist* or RING1B in XEN cells, TS cells, EpiLCs and differentiating EpiLCs. Immuno-RNA FISH detecting ubH2AK119 (Rhodamine red) together with RING1B or *Xist* (FITC) on TS cells, XEN cells, EpiLCs and differentiated EpiLCs.

RING1B-*Xist* immuno-RNA FISH indicated clear Xi associated accumulation of RING1B in TS but not XEN cells, whereas this analysis failed for EpiLCs and differentiated EpiLCs. For XEN cells, EpiLCs and differentiated EpiLCs we therefore performed double immunohistochemistry of RING1B and ubH2AK119, as ubH2AK119 as the catalytic product of this enzyme is the best marker for the Xi in XEN, EpiLC and differentiated EpiLC (see data below). This approach indicated that there was no clear accumulation of RING1B on the Xi in XEN cells, EpiLCs and differentiated EpiLCs; although sporadically accumulation could be observed (Fig 7). UbH2AK119, however, could be observed in all analysed cell lineages (Figs 4 and 7), again indicating that the detection of Xi associated enzyme complexes is not a requirement for the maintained presence of a corresponding chromatin modification. Similarly to H3K27me3, an immunostaining of ubH2AK119 in differentiated EpiLCs detected a higher percentage of cells presenting the modification.

### Accumulation of other heterochromatic markers

**Fig 8:**
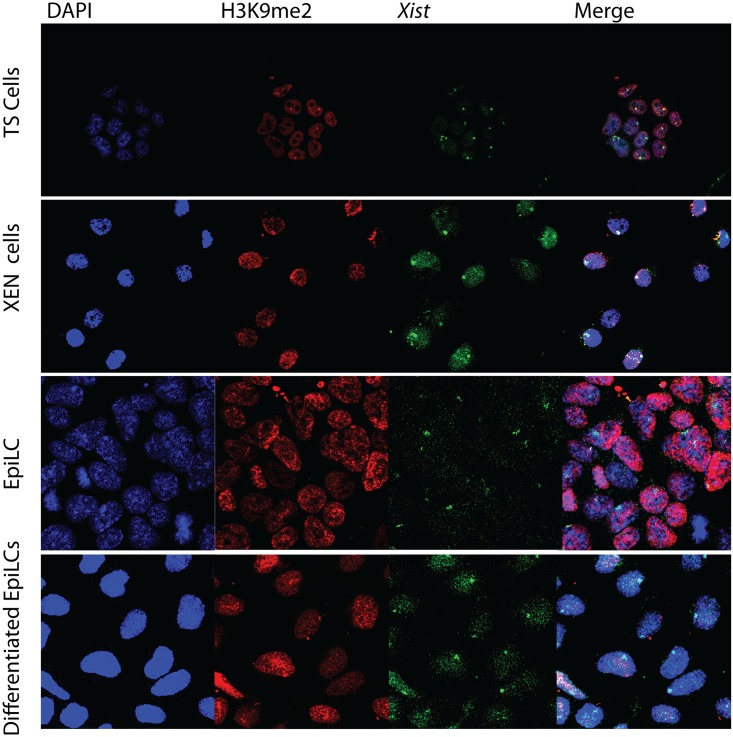
Immuno-RNA FISH for H3K9me2 and *Xist* in XEN cells, TS cells, EpiLCs and differentiating EpiLCs. Immuno-RNA FISH detecting H3K9me2 (Rhodamine red) together with *Xist* (FITC) on TS cells, XEN cells, EpiLCs and differentiated EpiLCs.

**Fig 9:**
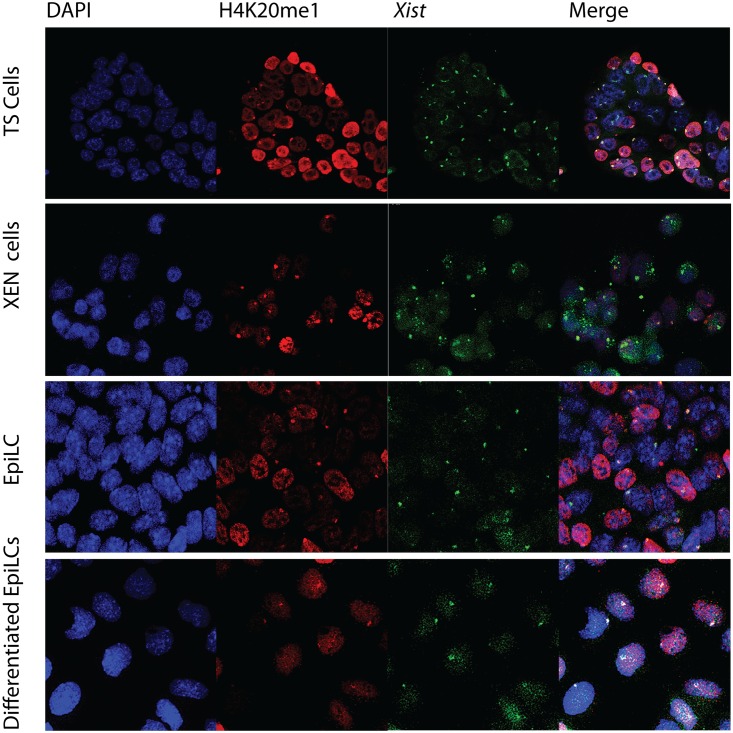
Immuno-RNA FISH for H4K20me1 and *Xist* in XEN cells, TS cells, EpiLCs and differentiating EpiLCs. Immuno-RNA FISH detecting H4K20me1 (Rhodamine red) together with *Xist* (FITC) on TS cells, XEN cells, EpiLCs and differentiated EpiLCs.

**Fig 10:**
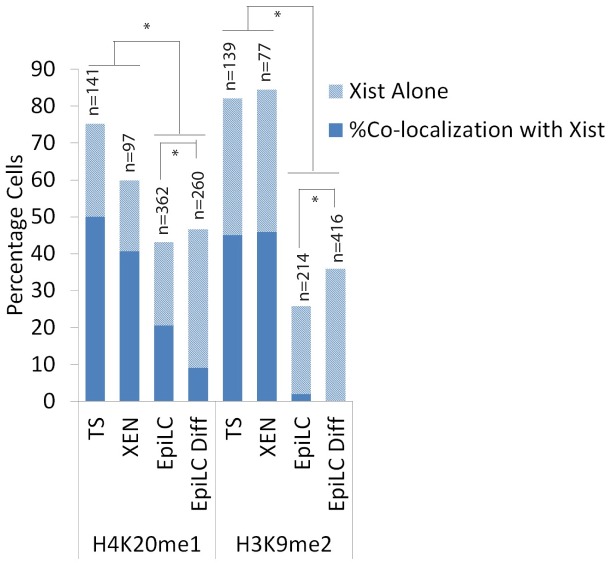
Percentage cells accumulating either *Xist* alone or showing *Xist* together with an accumulation of histone modifications H4K20me1 and H3K9me2 in TS cells, XEN cells, EpiLCs and differentiated EpiLCs. Percentages displayed in bar chart with sample size (n). Statistical significance (p<0,05) tested via z-test for proportion independent groups and shown in Figure.

**Fig 11:**
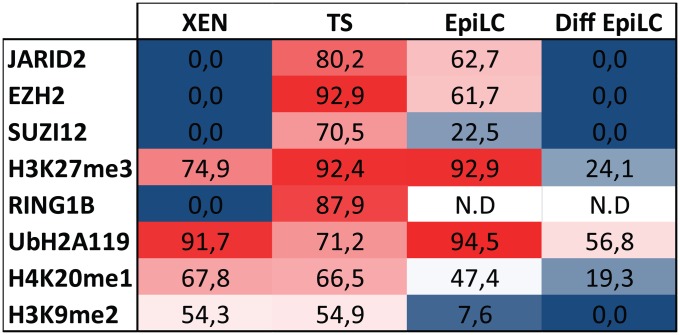
Percentage cells presenting heterochromatic modifications along *Xist* in XEN cells, TS cells, EpiLCs and differentiating EpiLCs. Percentage illustrated by a colour scale from no (blue) to complete (red) association between *Xist* and heterochromatic modifications.

H3K9me2 and H4K20me1 are both histone modifications associated with the Xi but not as well characterized as H3K27me3 and ubH2AK119. Several lysine 9 histone H3 methyltransferases, euchromatic and heterochromatic, have been characterized but it is unclear which enzyme catalyzes this modification on the Xi. We found that H3K9me2 accumulation, associating with *Xist* clouds, could only be observed in XEN and TS cells, but was not as abundant as H3K27me3 and ubH2AK119 (Fig 8). Immuno-RNA FISH indicates that H4K20me1, likely catalysed by SET8/PR-Set7 [24,25], is a better marker for the Xi than H3K9me2 as it was not only accumulated on the Xi in TS and XEN cells but,also accumulates to a minor extent on the Xi in EpiLCs (Figs 9, 10 and 11). In conclusion, we found that accumulation of PRC1 and PRC2 is very variable on the Xi, but that the modifications, ubH2A119 and H3K27me3, catalysed by these complexes are better Xi associated chromatin markers compared to H3K9me2 and H4K20me1.

## Discussion

**Fig 12:**
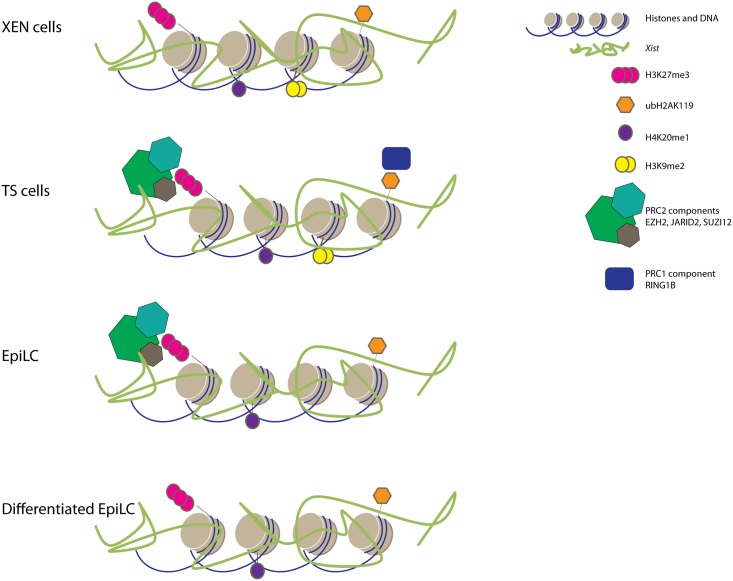
Overview of Xi associated heterochromatic marks in TS cells, XEN cells, EpiLCs and differentiated EpiLCs. Comprehensive overview of accumulation of RING1B, EZH2, SUZI12, JARID2, H4K20me1, H3K9me2, H3K27me3 and ubH2AK119 (categorized present if found in more than 10% of the cells).

Random and imprinted XCI are respectively depicted as stable and unstable. Since silencing of the X-chromosome relies on histone modifications, we tried to understand whether the stability of XCI could be simply conveyed to the presence or absence of histone modifications associated with the Xi. To address this question, reported chromatin modifications (Tables 1 and 2) associated with the Xi were assessed in all early cell lineages presenting either iXCI or rXCI. XEN and TS cells representing cell lineages with iXCI, whereas undifferentiated and differentiated EpiLC portrayed lineages with rXCI. This is the first study that provides a comprehensive overview of such a wide range of chromatin modifications associated with the Xi in different embryonic and extra-embryonic cell types. (Fig 12).

Upon ES cell differentiation, among the first epigenetic changes detectable after *Xist* accumulation are the loss of euchromatic features including RNA polymerase II, H3K9Ac, H4Ac, H4K16Ac. Our study indicates that this loss of euchromatic marks is a general characteristic of the Xi in a large percentage of cells in all lineages examined, and is central to the iXCI and rXCI processes. Recently, SPEN family members and co-factors have been identified as interactors of *Xist*. SPEN mediated recruitment of HDAC3 has been implicated in triggering histone deacetylation in differentiated ES cells [26,27,28,29,30]. Whether iXCI also involves SPEN mediated recruitment of HDAC3, and if H3K4 demethylases are actively recruited to the Xi to trigger the loss of RNA polymerase II needs further investigation [31].

The loss of active histone marks observed during ES cell differentiation is soon followed by the accumulation of histone modifications associated with facultative heterochromatin. In contrast to the absence of euchromatic marks we found that these heterochromatic features associated with the Xi were very heterogenic (Fig 12). Although H3K27me3 could be detected in all cell types, the PRC2 subunits EZH2, SUZ12 and JARID2, catalysing this modification, were only present on the Xi in both TS cells and EpiLCs. Similarly, ubH2AK119 accumulation associated with the Xi, could be detected in all cell types, despite the absence of Xi associated RING1B in XEN cells and undifferentiated and differentiated EpiLCs. The failure to detect PRC members in some tissues in spite of the presence of their catalysed histone modifications does not exclude accumulation of the PRC complexes, which may be present under the technical detection level, or the tested proteins may be substituted with other paralogs such as EZH1 [32] and RING1A [33]. The results, however, imply that a visible accumulation of PRC complexes on the Xi are merely an extrapolation of the plasticity of the genome of EpiLCs (rXCI) and TS cells (iXCI) rather than whether XCI is random or imprinted.

XCI in extra-embryonic tissues is classified as unstable in contrast to XCI in embryonic lineages. This is highlighted by the fact that forced expression of *Tsix*, a negative regulator of *Xist* and leading to *Xist* downregulation, results in reactivation of the Xi in extra-embryonic tissues [34]. Interestingly, also EpiLCs still have the capacity to initiate rXCI upon differentiation [35], a feature that is lost upon differentiation of this cell type, highlighting the plasticity of this cell type with respect to XCI. The presence of such a meta-stable state of XCI in these three cell types might be attributed to reduced levels of CpG methylation. In trophoblast and XEN cells CpG methylation is reported to be very low in comparison to embryonic tissues [36,37]. Interestingly, despite high expression levels of *Dnmts* [36], the DNA methylation level of CpG islands on the X-chromosome in female EpiLCs is also very low [36], and CpG islands only become methylated upon EpiLC differentiation (R.G. Boers, and J Gribnau personal communication). Therefore, the strong accumulation of H3K27me3, and possibly H4K20me1, and ubH2AK119 therefore might antagonize or precede the accumulation of DNA CpG methylation, reminiscent of findings showing a switch in the transcriptional control of repressed promoters via from H3K27me3 to DNA methylation during ES cell differentiation and carcinogenesis [38,39,40].

This study describes many undocumented chromatin modifications on the Xi in XEN cells, *in vitro* derived EpiLCs and differentiated EpiLCs. Most of our findings fit very well with documented observations, although we also found some noticeable exceptions. For instance, the stable association of H3K27me3 on the Xi in XEN cells contrasts previous findings [19,20]. Also, the failure to demonstrate a strong accumulation of H3K9me2 on the Xi in EpiLCs and differentiated EpiLCs is in conflict with earlier described studies in embryonic lineages [7,8,9,10]. This discrepancy might be explained through differences in detection efficiency, but could also be related to differences in cell types that have been used.

## Conclusion

In conclusion, what governs stability of XCI cannot be simply determined by the assessment of the presence or absence of histone modifications or classified depending on whether XCI is random or imprinted. XCI is initiated and maintained by a multitude of epigenetic layers consisting of histone modifications and DNA CpG methylation. The deposition and maintenance of histone modifications on the Xi is *Xist* dependent whereas the accumulation of DNA CpG methylation makes XCI maintenance irreversible and *Xist* independent. In a specific epigenetic context, XCI is relatively stable regardless whether XCI is random or imprinted. Problems in XCI stability may arise when XCI maintenance is still *Xist* dependent and the changing epigenetic context during differentiation and development cannot be implemented fast enough to create other epigenetic layers on the Xi. Which heterochromatic chromatin modifications can be found on the Xi is dependent on the epigenetic context and plasticity of the entire genome of the assessed cell. Despite the observed differences in histone modifications associated with the Xi in different lineages, our results indicate that both ubH2AK119 and H3K27me3 are the best heterochromatic markers for the Xi in all assessed lineages.

## Materials and Methods

### Embryo collection and cell culture

#### Ethics statement and embryo collection

All animal experiments were approved by the Erasmus MC Animal Experimental Commission and were in accordance with the legislation of animal experimentation in the Netherlands. Housing and husbandry of mice was provided by the animal experimentation center (EDC) at the Erasmus MC. Except for the TS cells, all analysed lineages were hybrid, derived from embryos from crossings of 129S2/SvHsd (Envigo, The Netherlands) or C57BL/10ScOlaHsd (Envigo, The Netherlands) female mice with Cast/Eij (Jackson, USA) males. The analysed TS cells were both hybrid and inbred 129S2/SvHsd (Envigo, The Netherlands). The ovarian cycle of females was synchronized by intra-peritoneal injections of Folligonan and Chorullon (both 150 μl, 50U/ml, both one injection with 48 hours in between). Following the last injection each female mouse was mated with a male mouse. About 20 hours after the last injection, mating was evaluated by looking for the presence of a white plug in the vagina. Females that had mated were euthanized by cervical dislocation at E3.5 for ES or XEN cell derivation or killed by cervical dislocation at E6.5 for the creation of TS cells.

#### XEN cell line derivation and culture conditions

E3.5 embryos were flushed with M2 medium and placed in M16 medium at 37°C and 5% CO2 before being placed in XEN derivation culture conditions. For XEN derivation, E3.5 embryos were placed into gelatinized wells covered with MEFs and cultured in XEN-medium (RPMI 1640; with 20% FBS, 1 mM sodium pyruvate, 2 mM L-glutamine, PS and 100μ M β-mercaptoethanol). Every 1–2 days, fresh medium was added until embryos were attached to the gelatinized surface of the well. After the embryos were attached and showed significant outgrowth, the XEN cells were split with 0.25% trypsin/EDTA and cultured in standard XEN culture conditions. For combined immunohistochemistry RNA-FISH, cells were grown onto plastic slide chamber flasks.

#### TS cell line derivation and culture conditions

TS cells were derived from E6.5 embryos. The extra embryonic part of E6.5 egg cylinders were plated in six-well plates on irradiated MEFs and maintained in XEN-medium supplemented with 25 ng/ml human recombinant fibroblast growth factor 4 (hrFGF4) and heparin sulfate. After the embryos were attached and there was significant outgrowth, the TS cells were split with 0.25% trypsin/EDTA and further cultured in standard TS culture conditions. For combined immunohistochemistry RNA-FISH, cells were grown onto plastic slide chamber flasks.

#### ES derivation and culture conditions

For ES derivation, E3.5 blastocysts were placed into culture dishes coated with gelatin (0.2%) and irradiated MEFs in ES cell medium containing DMEM, 15% fetal calf serum (FCS), PenStrep (PS), 1 mM non-essential amino acids (NEAA), 50mM β-mercaptoethanol, leukaemia inhibitory factor (LIF), MEK inhibitor (PD98059, 4μM) and GSK3inhibitor (CHIR99021, 3.3 μM). Approximately one week after embryo recovery the outgrowth of the ICM was enzymatically split and plated in the same culture conditions as previously described. After a few passages, inhibitors for MEK and GSK3 were removed from the culture medium.

#### ES differentiation towards EpiLC

ES cells were trypsinized with 0.25% trypsin/EDTA and cultured in EpiLC conditions [41] for 3 passages (with collagenase) before being analysed. For combined immunohistochemistry RNA-FISH, cells were grown onto plastic slide chamber flasks.

#### EpiLC differentiation towards mesoderm

EpiLC cells were for combined immunohistochemistry RNA-FISH cells passaged onto plastic slide chamber flasks and grown for an extra 48 to 72 hours in EpiLC medium devoid of IWP2 put in the presence of WNT3 and BMP4.

### Immunohistochemistry and RNA-FISH

#### DNA Xist probe

The *Xist* probe used was a 5kb cDNA BglII fragment covering exons 3–7 [42]. The probe was directly labeled by random priming. A total of 500ng DNA was dissolved in a total volume of 23 μl. 20μl of random primers 2.5x was added and denaturation was then performed at 95°C for 5 min. Immediately following the denaturation, the probe was cooled on ice and dNTPs, labeled dUTP and Klenow fragment were added. This mix was incubated at 37°C for 2 hours. For precipitation, Cot1-DNA, salmon sperm (SS) DNA, tRNA, sodiumacetate 3M and EtOH 100% were added to the labelled DNA. The mixture was frozen for 20 min in -20°C. To obtain the DNA pellet, the tube was centrifuged at 13200 rpm for 20–30 min and the supernatant was carefully removed. The pellet was thoroughly resuspended in 70% EtOH by vortexing and centrifuging at 13200 rpm for 5–10 min. Supernatant was carefully removed and the pellet was air-dried for 10 min. The labeled probe was dissolved with 50+ Hybridization mix and stored at -20°C.

#### Antibodies

Specifications of the utilized antibodies can be found in Table 3.

**Table 3:** Antibody pecifications.

Antibody	Dilution	Host	Supplier
H3K27me3	1:500	Rabbit	Diagenode CS-069-100
H4K20me1	1:500	Rabbit	Abcam 16974
H3K9me2	1:200	Mouse	Cosmo Bio (MCA-MABI0007-20-EX)
ubH2A	1:200	Rabbit	Cell signalling (8240)
H3K4me2	1:1500	Rabbit	Upstate 07–030
H3K9ac	1:1000	Rabbit	Sigma H9286
H4K16ac	1:100	Rabbit	Abcam 1240–100
H4ac	1:100	Rabbit	Upstate 06–598
RNApolII	1:600	Mouse	Abcam 817–100
RING1B	1:50	Mouse	Generous gift from Dr. H. Koseki
SUZ12	1:100	Rabbit	Diagenode pAb-029-050
EZH2	1:200	Rabbit	Leica Microsystems (NCL-L-EZH2)
JARID2	1:500	Rabbit	Abcam (48137)

#### RNA FISH

Cells cultured on plastic slide chamber flasks were fixed in 4% paraformaldehyde (PFA) for 10 min at room temperature and subsequently 3 times rinsed in PBS. The coverslips were incubated with 0.2% pepsin dissolved in water and incubated at 37°C in a water bath. After 4 min exposure, the pepsin was removed and the coverslips were rinsed in water. The coverslips were post-fixed in 4% PFA for 5 min and again 3 times washed with PBS. To dehydrate the cells, an ethanol gradient was used with 70%, 90% and 100% EtOH. Cells were hybridized overnight at 37°C with the denatured *Xist* probe (10min at 99°C followed by 45min at 37°C). The next day the coverslips were 3 times washed with 0.05x Saline-Sodium Citrate (SSC) in a pre-heated water bath at 40°C. The cells were mounted with Vectashield/DAPI.

#### RNA FISH and immunohistochemistry

Cells cultured on plastic slide chamber flasks were fixed in 3% PFA for 10 min at room temperature and 3 times rinsed in PBS. Permeabilization was performed with PBS containing 20% Triton-X 100 and 20 μM Vanadyl Ribonucleoside Complex (VRC)(New England Biolabs S1402S). After rinsing 3 times in PBS, preparations were blocked in a blocking solution containing bovine serum albumin (BSA) (Biolabs, B90015) and 20 μM VRC in PBS for 30 min. The primary antibody, diluted in blocking solution, was applied to the wells of the slide chamber flasks and incubated in a humid box for 1 hour at room temperature (Table 3). After 3 times 10 min washes with PBS, the secondary antibody, diluted in the blocking solution was applied to the wells of the slide chamber flask and incubated for 1 hour at room temperature.

The secondary antibody, was removed by 3 washes in PBS for 5min each. Following removal of the plastic chamber, the remaining plastic slides were accordingly post-fixed in 4% PFA for 10 min at room temperature, rinsed in PBS and washed two times with 2x SSC before being air dried. The denatured *Xist* probe was applied on the slides, coverslips were placed and glued with rubber cement onto the slide and incubated for 15–20 hours in a dark box at 37°C. After 3 washes with 0.05x SSC at 38–40°C, DNA was counterstained for 2 minutes in 10μl DAPI. A Leica TCS SP 5 confocal microscope and Adobe Photoshop CS 6 and Illustrator were used for image acquisition.

## Supporting Information

S1 Fig Loss of euchromatic marks on Xi in TS cells. Immuno-RNA FISH on TS cells stained for euchromatic histone modifications (Rhodamine red) along *Xist* RNA (FITC).
(TIF)

S2 Fig Loss of euchromatic marks on Xi in XEN cells. Immuno-RNA FISH on XEN cells stained for euchromatic histone modifications (Rhodamine red) along *Xist* RNA (FITC).
(TIF)

S3 Fig Loss of euchromatic marks on Xi in EpiLC cells. Immuno-RNA FISH on EpiLC cells stained for euchromatic histone modifications (Rhodamine red) along *Xist* RNA (FITC).
(TIF)

S4 Fig Loss of euchromatic marks on Xi in differentiated EpiLC cells. Immuno-RNA FISH on differentiated EpiLC cells stained for euchromatic histone modifications (Rhodamine red) along *Xist* RNA (FITC).
(TIF)

S5 Fig Accumulation of PRC2 complex members on Xi in TS cells. Immuno-RNA FISH on TS cells stained for PRC2 complex members JARID2 and SUZ12 (Rhodamine red) along *Xist* RNA (FITC).
(TIF)

S6 Fig No accumulation of PRC2 complex members on Xi in XEN cells. Immuno-RNA FISH on XEN cells stained for PRC2 members JARID2 and SUZ12 (Rhodamine red) along *Xist* RNA (FITC).
(TIF)

S7 Fig Accumulation of PRC2 complex members on Xi in EpiLC cells. Immuno-RNA FISH on EpiLCs stained for PRC2 complex members JARID2 and SUZ12 (Rhodamine red) along *Xist* RNA (FITC).
(TIF)

S8 Fig No accumulation of PRC2 complex members on Xi in differentiated EpiLC cells. Immuno-RNA FISH on differentiated EpiLCs stained for PRC2 complex members JARID2 and SUZ12 (Rhodamine red) along *Xist* RNA (FITC).
(TIF)
