# Supplementary figures and images for: Characterization of Histone Modifications Associated with Inactive X-Chromosome in Trophoblast Stem Cells, eXtra-Embryonic Endoderm Cells and in In Vitro Derived Undifferentiated and Differentiated Epiblast Like Stem Cells
Source: PLoS One. 2016 Dec 15;11(12):e0167154. doi: 10.1371/journal.pone.0167154 (PMC5157996; doi:10.1371/journal.pone.0167154)

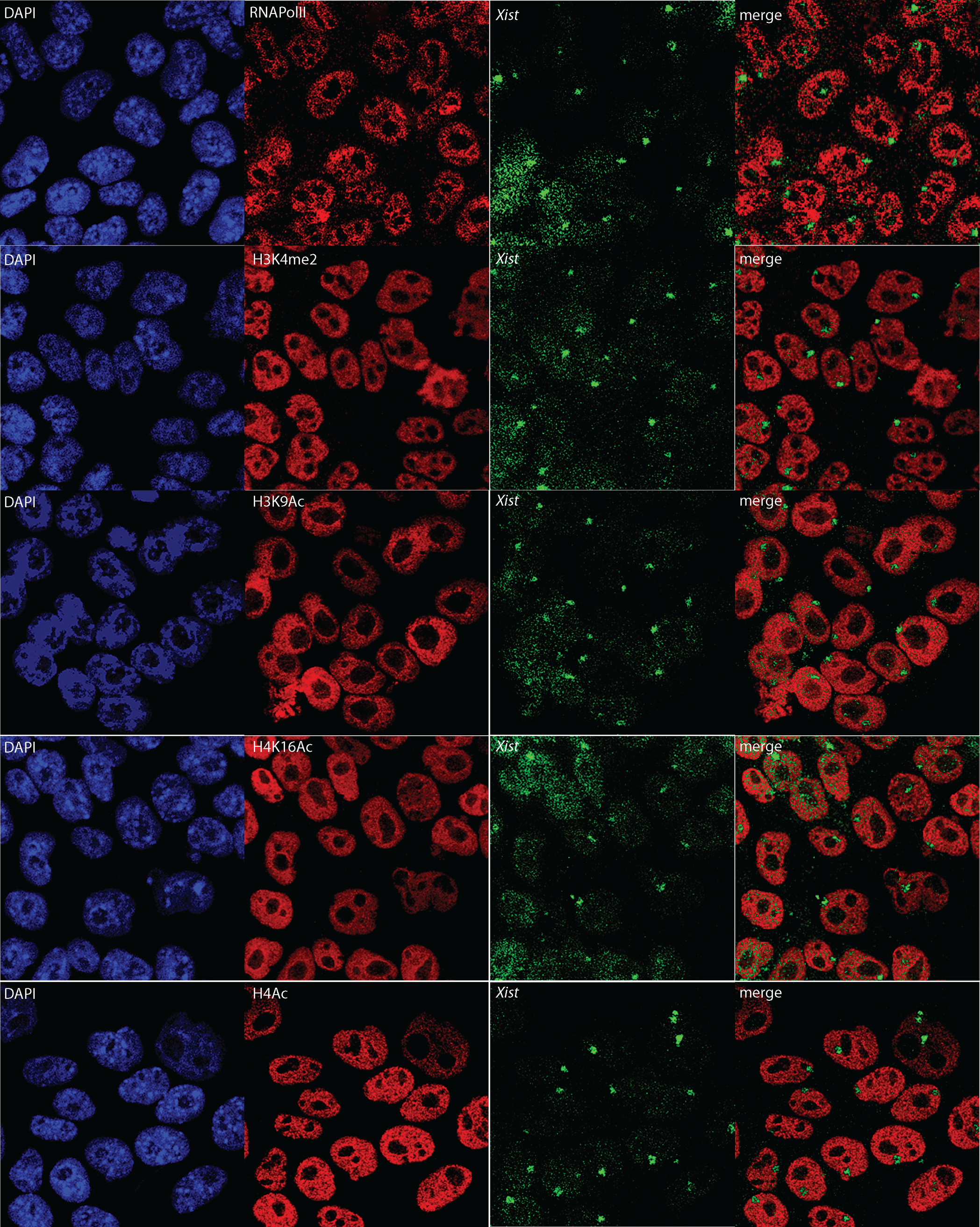

Supplement: S1 Fig — Immuno-RNA FISH on TS cells stained for euchromatic histone modifications (Rhodamine red) along Xist RNA (FITC). (TIF) [file pone.0167154.s001.tif]

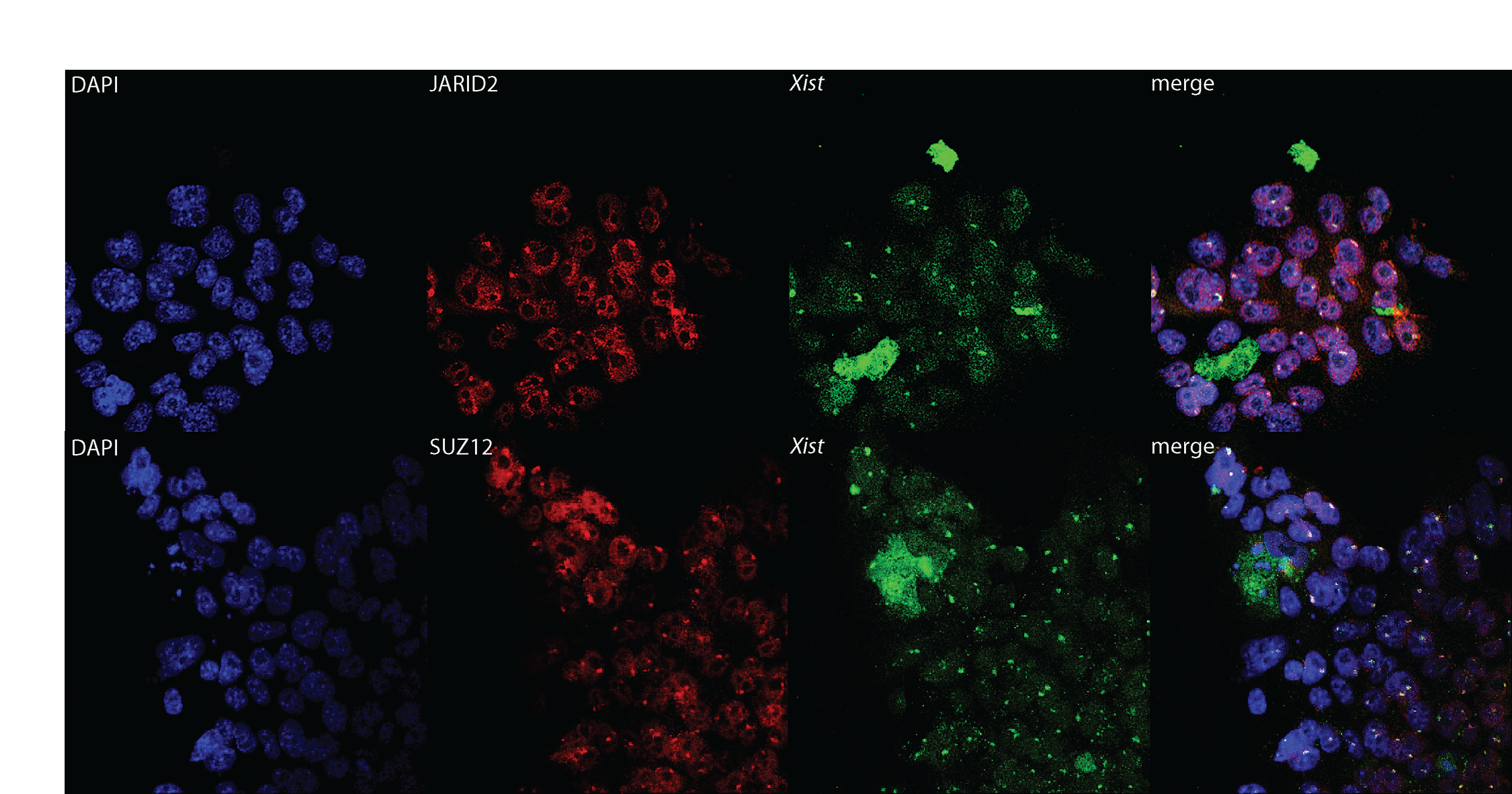

Supplement: S2 Fig — Immuno-RNA FISH on XEN cells stained for euchromatic histone modifications (Rhodamine red) along Xist RNA (FITC). (TIF) [file pone.0167154.s002.tif]

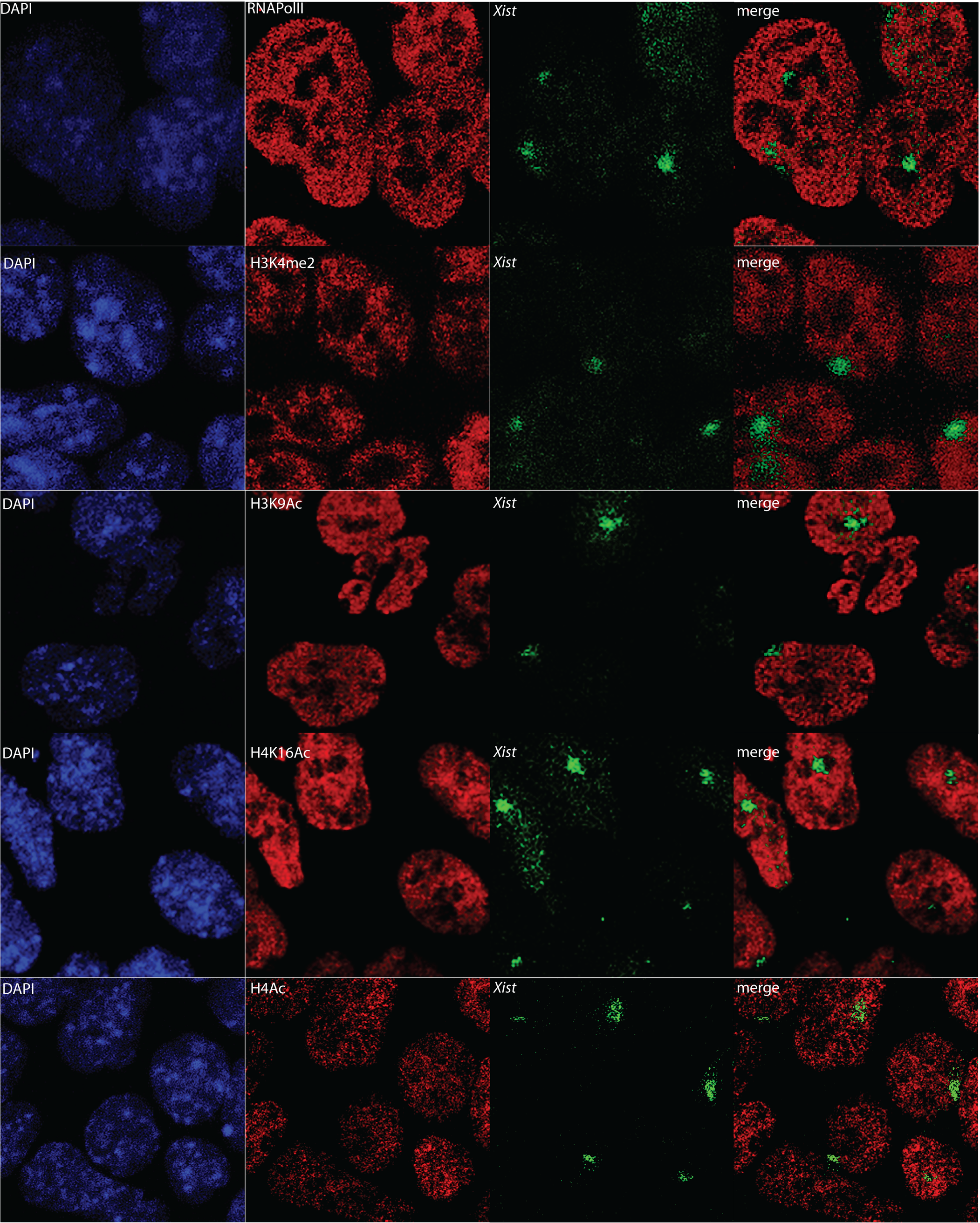

Supplement: S3 Fig — Immuno-RNA FISH on EpiLC cells stained for euchromatic histone modifications (Rhodamine red) along Xist RNA (FITC). (TIF) [file pone.0167154.s003.tif]

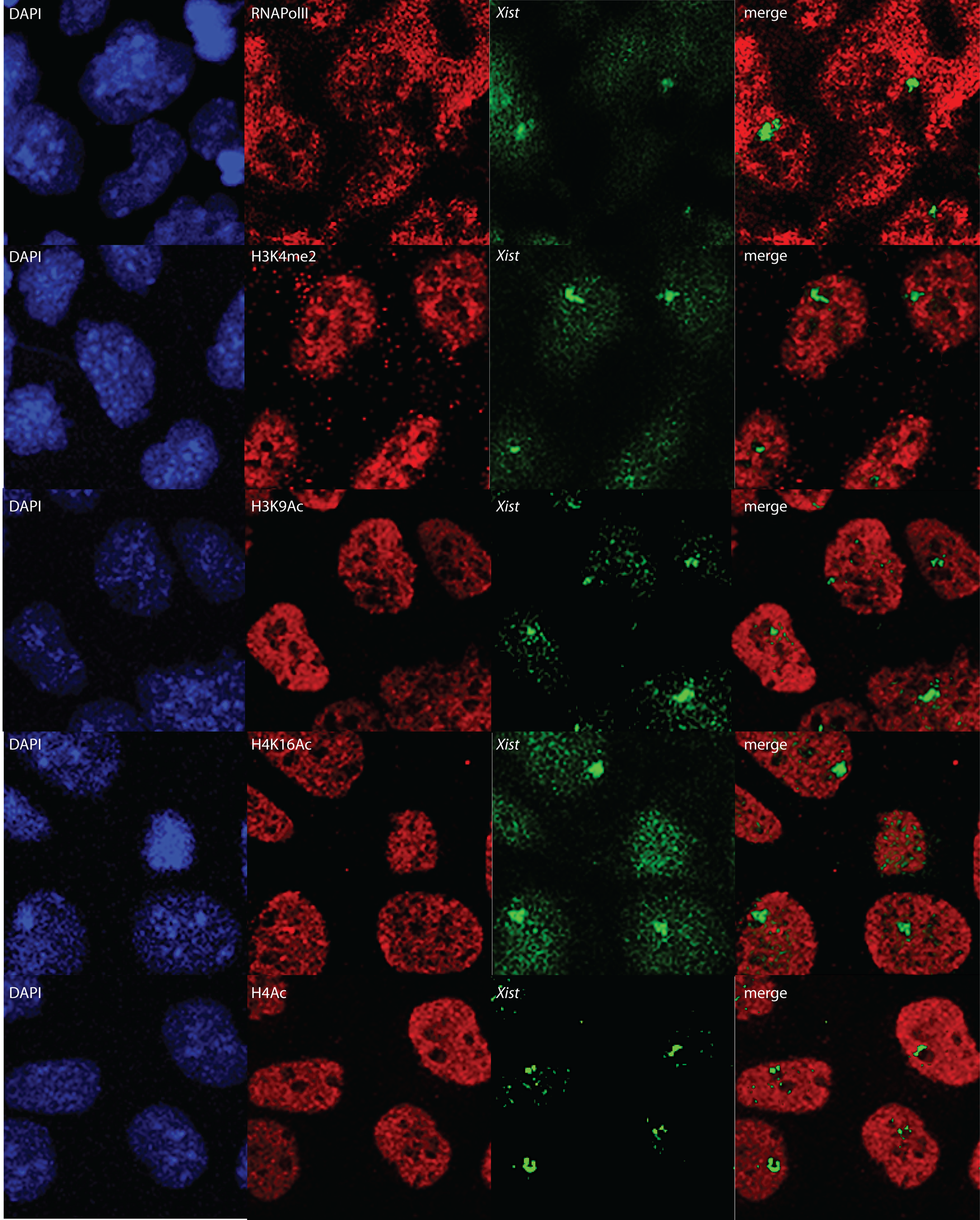

Supplement: S4 Fig — Immuno-RNA FISH on differentiated EpiLC cells stained for euchromatic histone modifications (Rhodamine red) along Xist RNA (FITC). (TIF) [file pone.0167154.s004.tif]

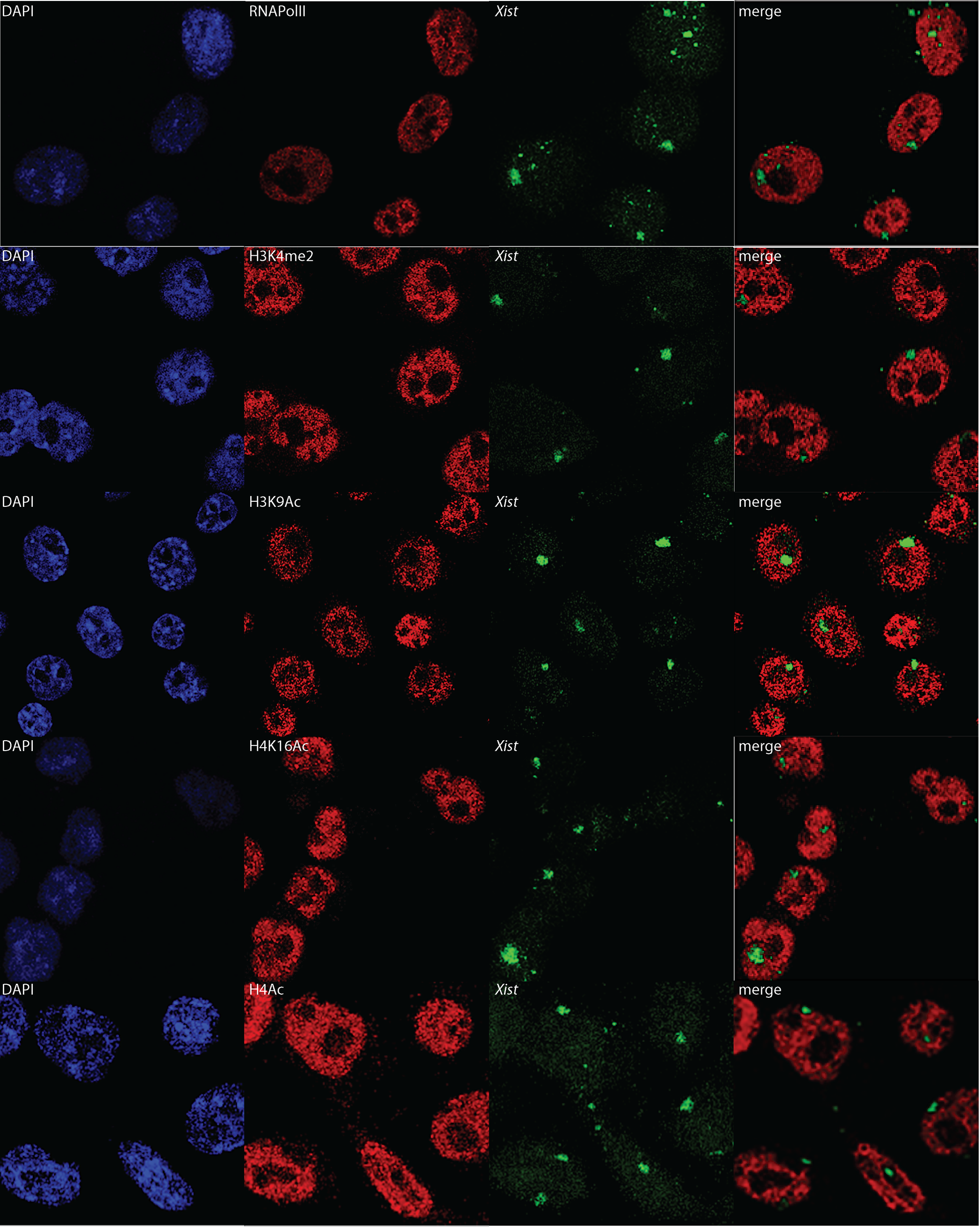

Supplement: S5 Fig — Immuno-RNA FISH on TS cells stained for PRC2 complex members JARID2 and SUZ12 (Rhodamine red) along Xist RNA (FITC). (TIF) [file pone.0167154.s005.tif]

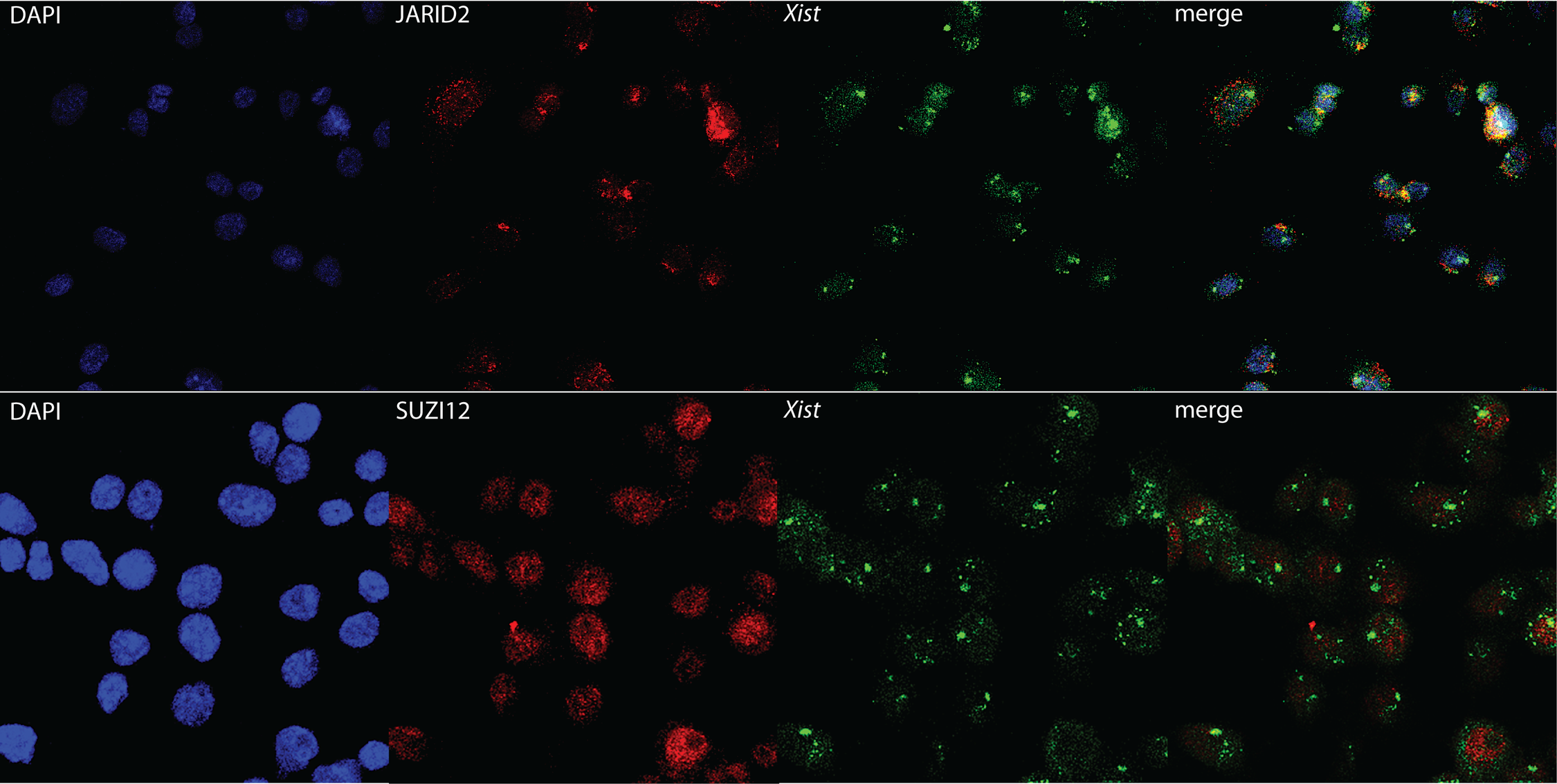

Supplement: S6 Fig — Immuno-RNA FISH on XEN cells stained for PRC2 members JARID2 and SUZ12 (Rhodamine red) along Xist RNA (FITC). (TIF) [file pone.0167154.s006.tif]

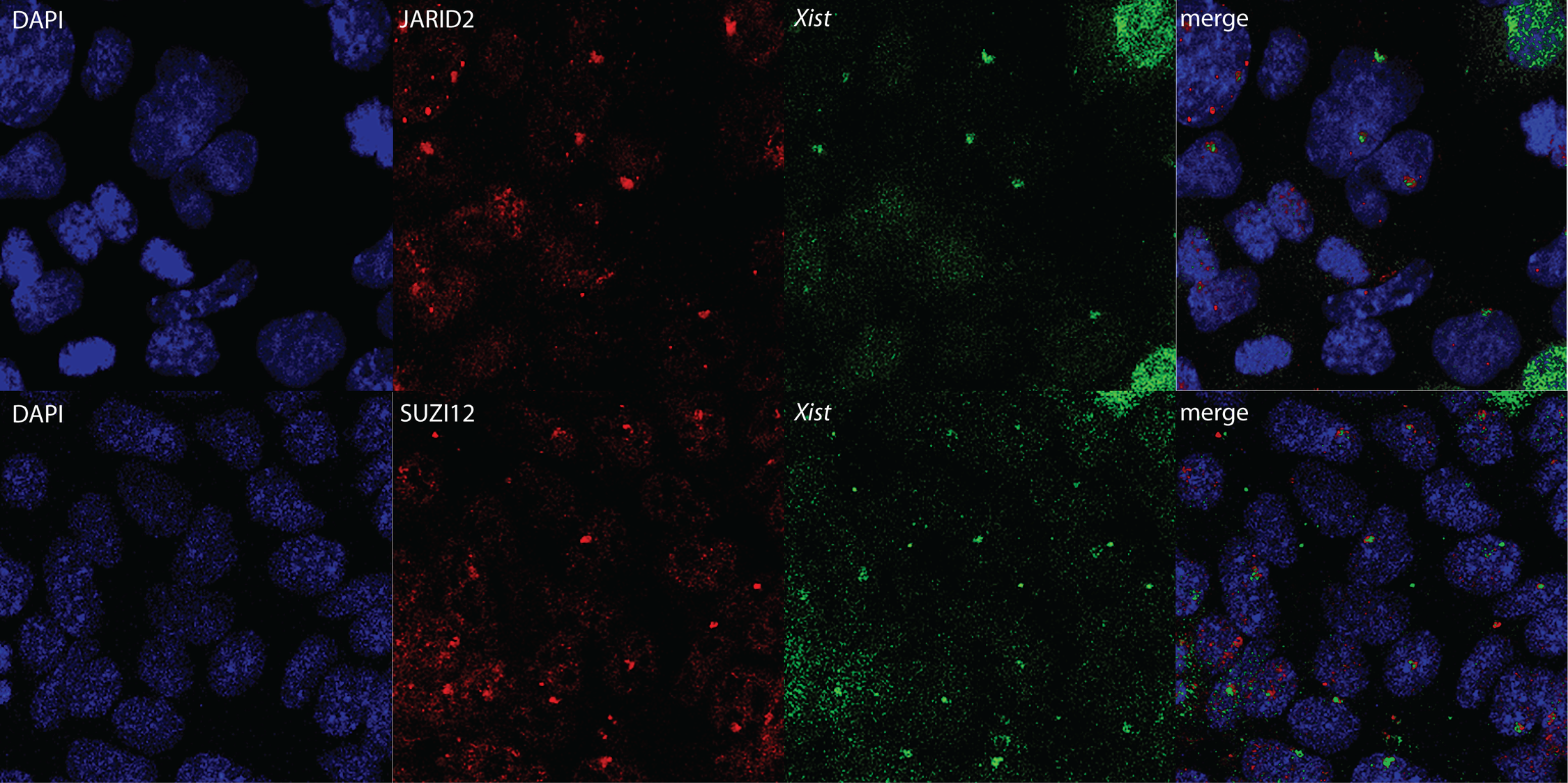

Supplement: S7 Fig — Immuno-RNA FISH on EpiLCs stained for PRC2 complex members JARID2 and SUZ12 (Rhodamine red) along Xist RNA (FITC). (TIF) [file pone.0167154.s007.tif]

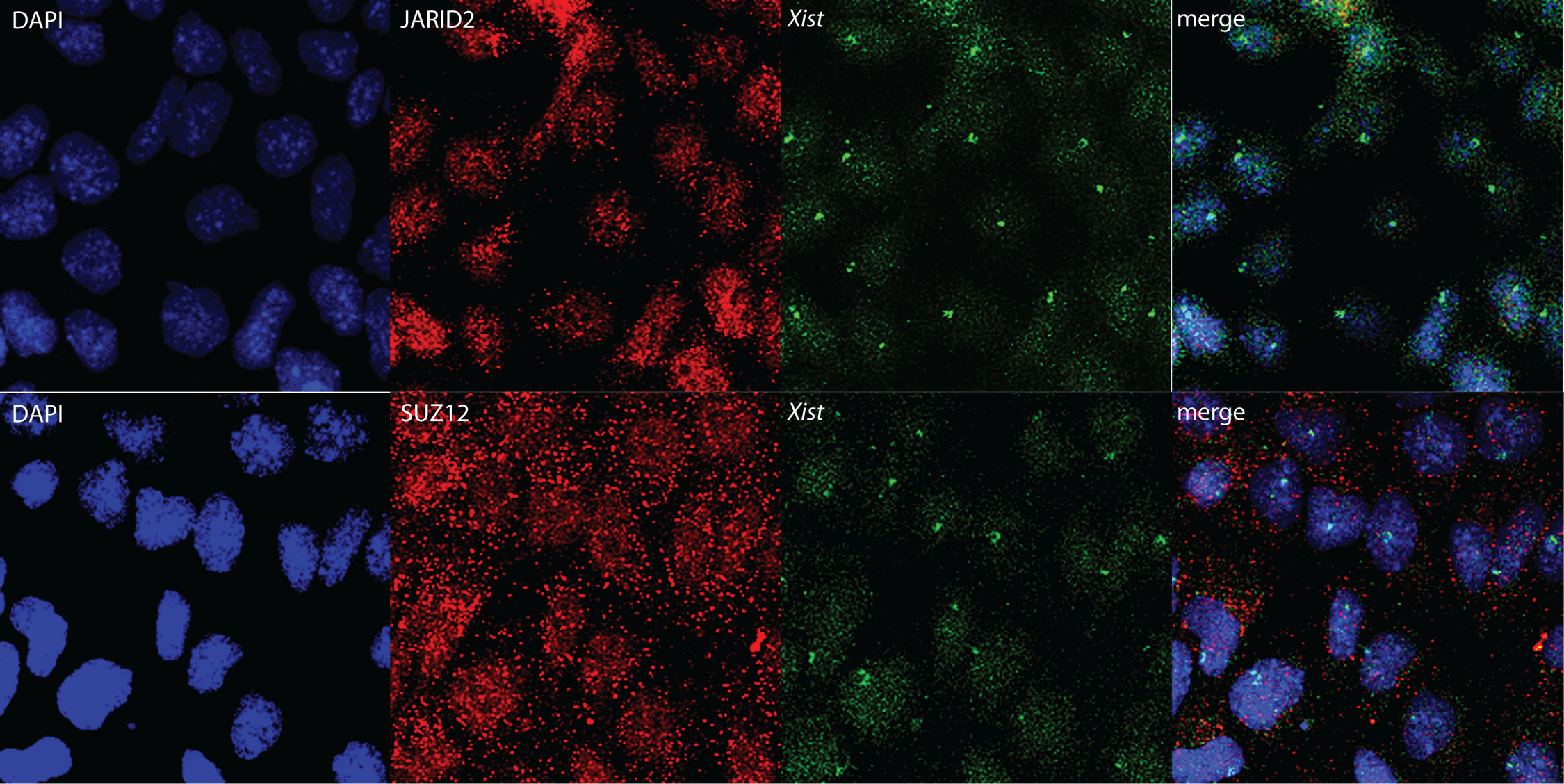

Supplement: S8 Fig — Immuno-RNA FISH on differentiated EpiLCs stained for PRC2 complex members JARID2 and SUZ12 (Rhodamine red) along Xist RNA (FITC). (TIF) [file pone.0167154.s008.tif]
